# Characterization of the GntR family regulator HpaR1 of the crucifer black rot pathogen *Xanthomonas campestris* pathovar *campestris*

**DOI:** 10.1038/srep19862

**Published:** 2016-01-28

**Authors:** Hui-Zhao Su, Liu Wu, Yan-Hua Qi, Guo-Fang Liu, Guang-Tao Lu, Ji-Liang Tang

**Affiliations:** 1 State Key Laboratory for Conservation and Utilization of Subtropical Agro-bioresources, The Key Laboratory of Ministry of Education for Microbial and Plant Genetic Engineering, and College of Life Science and Technology, Guangxi University, 100 Daxue Road, Nanning, Guangxi 530004, China

## Abstract

The GntR family transcription regulator HpaR1 identified from *Xanthomonas campestris* pv. *campestris* has been previously shown to positively regulate the genes responsible for hypersensitive reaction and pathogenicity and to autorepress its own expression. Here, we demonstrated that HpaR1 is a global regulator that positively regulates diverse biological processes, including xanthan polysaccharide production, extracellular enzyme activity, cell motility and tolerance to various stresses. To investigate the regulatory mechanisms of HpaR1, we began with xanthan polysaccharide production, which is governed by a cluster of *gum* genes. These are directed by the *gumB* promoter. Disruption of HpaR1 significantly reduced *gumB* transcription and an electrophoretic mobility shift assay demonstrated that HpaR1 interacts directly with *gumB* promoter. DNase I footprint analysis revealed that HpaR1 and RNA polymerase were bound to the sequences extending from −21 to +10 and −41 to +29 relative to the transcription initiation site of *gumB*, respectively. Furthermore, *in vitro* transcription assays showed that HpaR1 facilitated the binding of RNA polymerase to *gumB* promoter, leading to an enhancement of its transcription. These results suggest that HpaR1 regulates *gumB* transcription via a mechanism similar but different to what was found, until now, to only be used by some MerR family transcription activators.

*Xanthomonas campestris* pv. *campestri*s (*Xcc*) is a gram-negative bacterium that causes black rot, one of the most devastating diseases of vegetable brassica crops worldwide^1^. This pathogen can infect almost all the members of the crucifer family (Brassicaceae), including important vegetables such as broccoli, Brussel sprouts, cabbage, cauliflower, kale, mustard and radish, rape, and the model plant *Arabidopsis thaliana*. In addition to being an important plant pathogen, *Xcc* produces xanthan gum (extracellular polysaccharide, EPS), a biopolymer with great commercial significance. Xanthan gum is widely used in agriculture, petroleum production and in the food industry as a viscosifier, thickener, stabilizer, emulsifier and suspending agent^2^,^3^. Recently, various techniques have been developed to modify xanthan gum as a potential biopolymeric carrier for drug delivery systems^4^. Because of its agricultural and industrial importance *Xcc* is used as a model organism for studying the molecular mechanisms of bacterial EPS synthesis and phytopathogenicity^2^,^5^. The entire genome sequences of several *Xcc* strains have been determined^6^,^7^,^8^, and a large number of genes associated with pathogenicity and xanthan production have been identified^1^,^9^,^10^. Among these, the *gum* and *hrp* gene clusters are essential for xanthan synthesis and the hypersensitive response and pathogenicity, respectively. The *gum* cluster comprises 12 genes, *gumB* to *gumM*, which are mainly expressed as an operon that is regulated by a promoter upstream of the first gene, *gumB*^11^,^12^. The *hrp* cluster consists of six operons (*hrpA* to *hrpF*), whose expression is controlled directly or indirectly by several regulators such as HrpX, HrpG, HpaS, HpaR, HpaR1 and Zur^13^,^14^,^15^. HrpX is an AraC-type transcriptional activator, which activates the expression of all six operons in the *hrp* cluster^13^. HpaS and HrpG constitute a two-component signal transduction system that positively regulates the expression of *hrpX*^15^. HpaR is a MarR family transcriptional regulator, which is positively regulated at the transcriptional level by HrpX^16^. Zur is a key regulator for zinc homeostasis, which positively affects the expression of the *hrp* cluster via HrpX^13^. HpaR1 is a GntR family transcriptional regulator, which may regulate indirectly the expression of *hrpG*^14^.

GntR family members belong to the helix-turn-helix group of bacterial transcriptional regulators. Structurally, GntR members possess a conserved N-terminal winged helix-turn-helix DNA-binding domain and a diverse C-terminal signalling domain. Based on the topology and secondary structure of their diverse C-terminal signalling domains, GntR members have been further classified into seven subfamilies: AraR, DevA, FadR, HutC, MocR, PlmA and YtrA^17^. HpaR1 belongs to the YtrA subfamiliy^14^. Recently, we found that HpaR1 is a global regulator of diverse *Xcc* cellular processes. In this paper, we describe how HpaR1 is involved in the regulation of extracellular enzyme and xanthan production, cell motility, and the tolerance of *Xcc* to various stresses. Furthermore, we demonstrate that HpaR1 positively regulates xanthan production by binding to the *gumB* promoter region to facilitate the interaction between RNA polymerase and the promoter, leading to a transcriptional enhancement of the *gum* cluster of genes.

## Results

### HpaR1 regulates diverse cellular processes including extracellular enzyme activity, xanthan production, cell motility and tolerance to various stresses

**Figure 1:**
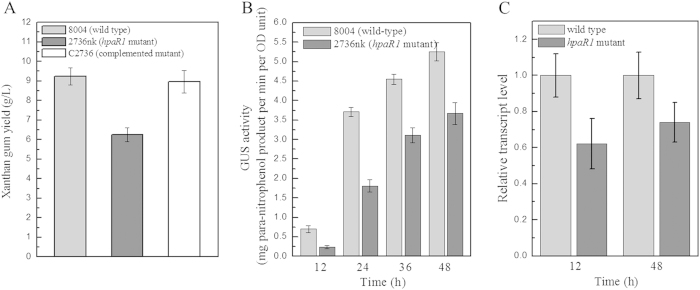
HpaR1 positively regulates xanthan gum production in *Xcc*. (**A**) The *hpaR1* mutant produced significantly less xanthan gum, compared to the wild-type strain. Mean weight of xanthum gum extracted from the HpaR1 mutant strain 2736nk, the wild type strain 8004 and the complemented strain C2736. (**B**) The expression of *gumB* is affected positively by HpaR1 in xanthum producing medium, as revealed by promoter-reporter analysis. The graph shows the mean ± standard deviation (n = 3) *gumB* promoter*-*GUS activity in the wild type strain 8004 compared to the *hpaR1* mutant strain 2736nk. The experiment was repeated twice, and similar results were obtained. (**C**) The expression of *gumB* is positively regulated by HpaR1, as revealed by qRT-PCR analysis of the transcription of *gumB* in *Xcc* wild type and *hpaR1* mutant strains. Values given are the means ± standard deviations of triplicate measurements from a representative experiment; similar results were obtained in two other independent experiments.

**Figure 2:**
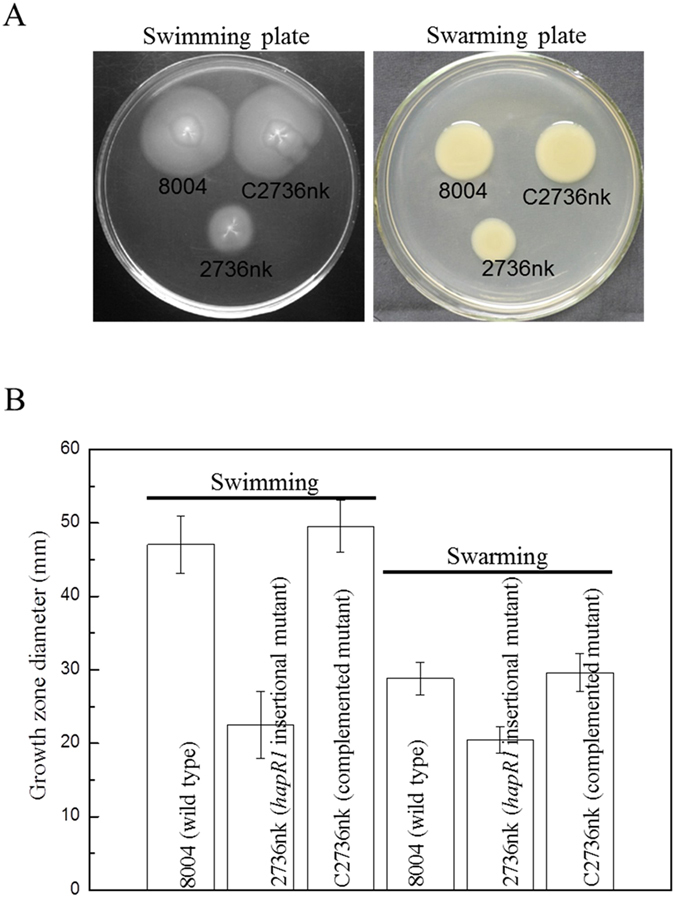
HpaR1 regulates positively the motility of *Xcc*. (**A**) Example photographs of *Xcc* wild type strain 8004, *hpaR1* mutant strain 2736nk and complemented strain C2736 stabbed into ‘swim’ (0.28% agar) medium followed by incubation at 28 °C for 4 days and inoculated onto ‘swarm’ (0.6% agar) medium followed by a 3-day incubation at 28 °C. (**B**) Mean (plus and minus standard deviation) measurements of colony diameters of each strain on the different media.

**Figure 3:**
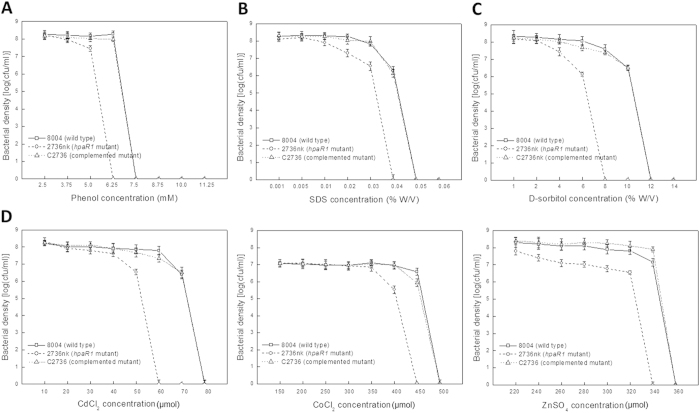
HpaR1 is involved in the tolerance of *Xcc* to phenol, SDS, D-sorbitol and heavy metal cations. Cultures of *Xcc* strains were diluted and plated on an NYG plate supplemented with different concentrations of phenol (**A**) SDS (**B**) D-sorbitol (**C**) and heavy metal salts (CoCl_2_, CdCl_2_ and ZnSO_4_) (**D**). Bacterial colonies were counted after incubation at 28 °C for 3 days. The representative results of only one out of three replicated experiments are presented.

As mentioned above, our previous work demonstrated that the GntR family member HpaR1 regulates the hypersensitive response (HR) and virulence of *Xcc*^14^. To further investigate whether HpaR1 is involved in other cellular processes, we examined the activity of extracellular enzymes, xanthan production, biofilm dispersal, cell motility and stress tolerance of the *hpaR1* mutant, which all contribute to the full virulence of *Xcc*. *Xcc* produces a range of extracellular enzymes, including amylase, endoglucanase, pectate lyase and protease. As shown in Table 1, the activities of all these enzymes produced by the *hpaR1* mutant strain 2736nk were significantly diminished compared to the wild-type strain 8004 (*P* = 0.01 by *t* test). Similarly, the xanthan production and cell motility of the mutant were also significantly reduced (Figs 1A and 2). The tolerance of the mutant to phenol, D-sorbitol, SDS and heavy metal salts (CoCl_2_, CdCl_2_ and ZnSO_4_) was also determined. The results showed that the tolerance of the mutant cells to all of the stresses tested was significantly reduced (Fig. 3). As we know the wild-type strain 8004 does not form a biofilm in L medium as it produces endo-β-1,4-mannanase, which contributes to bacterial aggregate dispersal^18^, we also tested the cell aggregate formation of the *hpaR1* mutant in L medium. The result displayed that similar to the wild type, the mutant did not form aggregates (data not shown). This indicates that although HpaR1 regulates the production of extracellular enzymes and polysaccharides, some of which can influence aggregative behaviour^18^, mutation of *hpaR1* did not lead to an aggregated mode of growth. Overall, these results reveal that *Xcc* HpaR1 is a global regulator that regulates diverse cellular processes, including the production of extracellular enzymes and xanthan, and cell motility, as well as tolerance to various stresses.

### HpaR1 positively regulates the expression of the *gum* operon

It has been well demonstrated that the *gum* gene cluster is responsible for xanthan synthesis and export^8^,^11^,^19^. As described above, the *gum* cluster is expressed as an operon from a promoter upstream of the first gene, *gumB*^11^,^12^. To explore whether the xanthan reduction caused by the mutation in *hpaR1* was attributed to a decrease in the expression of the *gum* genes, a *gumB-gus* reporter plasmid for transcriptional analysis was constructed. A 546-bp DNA fragment harbouring the *gumB* promoter sequence was amplified by PCR and cloned into the plasmid pL6*gus*, which harbours the promoterless *gusA* gene in the *Bam*HI/*Sph*I sites of pLAFR6. The resulting reporter plasmid pGUSgum was introduced by triparental conjugation into the *hpaR1* mutant 2736nk and the wild-type strain 8004. The GUS activities produced by the obtained transconjugant strains 2736 nk/pGUSgum and 8004/pGUSgum cultured in xanthan production assay medium (NY medium containing 2% glucose) were then assessed. The GUS activity produced by strain 2736 nk/pGUSgum was reduced by 65.6%, 51.2%, 31.6% and 30.1% compared to strain 8004/pGUSgum when cultured for 12 h, 24 h, 36 h and 48 h, respectively (Fig. 1B). The expression level of *gumB* in the *hpaR1-*deficient mutant was further investigated by qRT-PCR analysis. The *hpaR1* mutant strain 2736nk and wild-type strain 8004 cultured under the same conditions as used for GUS activity determination were assayed. The result showed that the *gumB* transcript level in the mutant was decreased by 38% and 26% compared to the wild-type when grown for 12 and 48 h, respectively (Fig. 1C). Taken together, these results revealed that disruption of *hpaR1* led to a significant reduction in the expression level of *gumB*, indicating that HpaR1 positively regulates the expression of the *gum* operon.

### HpaR1 binds to 31 nucleotides in the *gumB* promoter region

**Figure 4:**
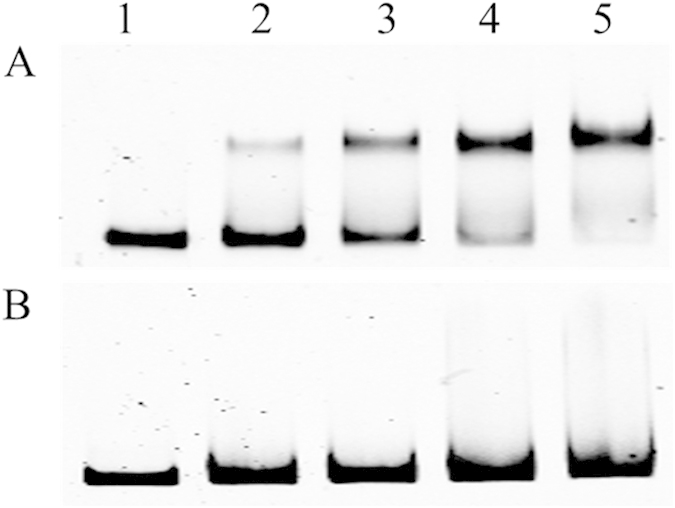
HpaR1 interacts directly with the *gumB* promoter, as revealed by an electrophoretic mobility shift assay. A FAM-labelled 529-bp DNA fragment of the *gumB* promoter (**A**) and 311-bp DNA fragment of the promoter of *XC_1045* (as a control) (**B**) were incubated with purified His_6_-HpaR1 protein for 20 min at 30 °C, respectively. Lanes 1–5, FAM-labelled DNA (1.0 nM) with 0, 5, 10, 15 and 20 nM of His_6_-HpaR1 protein, respectively.

To investigate whether HpaR1 directly regulates the expression of the *gum* gene cluster, we sought to detect if HpaR1 protein binds to the *gumB* promoter sequence using an EMSA. A His_6_-tagged HpaR1 protein was constructed and expressed in *E. coli* as described previously^14^. The binding ability of the purified His_6_-HpaR1 protein to a 529-bp DNA fragment encompassing the *gumB* promoter was then determined by EMSA. As shown in Fig. 4A, the His_6_-HpaR1 protein retarded the promoter sequence to a defined position. Equivalent experiments with a promoter fragment of an unrelated gene, specifically a 311-bp fragment containing the promoter of *XC_1045* that encodes a hypothetical protein, showed no binding (Fig. 4B). This suggests that the binding of HpaR1 to the *gumB* promoter was specific. These data reveal that HpaR1 directly interacts with the *gumB* promoter.

**Figure 5:**
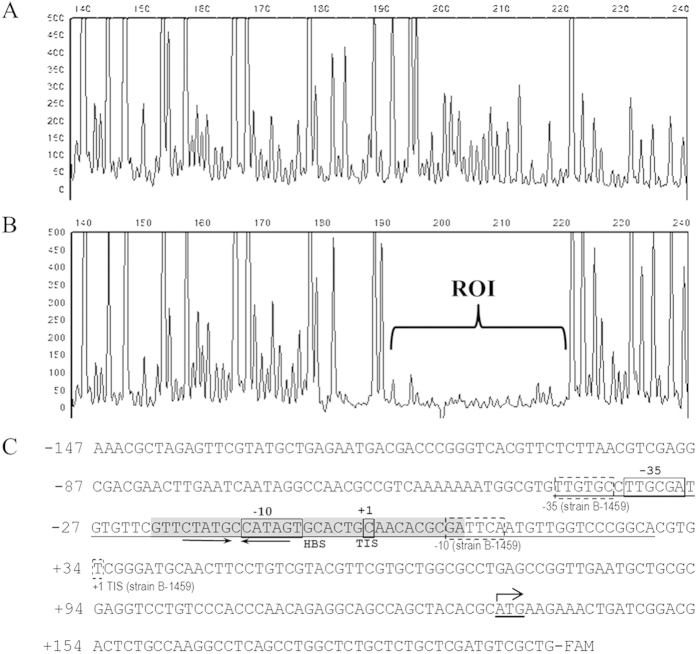
HpaR1 and RNA polymerase bind to 31 and 70 nucleotides in the *gumB* promoter region of *Xcc* strain 8004, respectively, as revealed by the dye primer-based DNase I footprint assays. Electropherograms show the protection pattern of the *gumB* promoter after digestion with DNase I following incubation in the absence (**A**) or presence (**B**) of 10 μM His_6_-HpaR1. ROI, region of interest. (**C**) The *gumB* promoter sequence of strain 8004 with a summary of the DNase I footprint assay results. The transcription initiation site (TIS) determined in strain 8004 (this work) and strain B-1459^11^ is indicated by the solid-line square and dash-line square, respectively. The −35 and −10 elements predicted in strain 8004 (this work) and strain B-1459^11^ are indicated by a solid-line rectangle and dash-line rectangle, respectively. The HpaR1 binding sequence (HBS) is highlighted in grey background. The dyad symmetrical sequence is indicated by arrows. The RNA polymerase binding region is underlined, which was also determined by a dye primer-based DNase I footprint assay (Supplementary Fig. 2).

To determine the precise HpaR1-binding sequence in the *gumB* promoter region, a dye primer-based DNase I footprint assay was performed. A 330-bp DNA fragment was amplified from the *gumB* promoter region by PCR with the primers Pgum-3F and gum-3R labelled fluorescently with 6-carboxyfluorescein (6-FAM) (Supplementary Table 1) and incubated with 10 μM His_6_-HpaR1 proteins before being digested with DNase I. After 5 min digestion, the reaction was terminated and the digestion pattern was examined on the 3730 DNA Analyzer. By comparing the electropherograms with and without His_6_-HpaR1 protein using GeneMarker software (Softgenetics), a specific His_6_-HpaR1-protected region within the *gumB* promoter was determined (Fig. 5A,B). As shown in Fig. 5C, the protected region consists of 31 nt, which spans from nt −54 to −24, relative to the transcription initiation site determined in the *Xcc* strain B-1459^11^. This region is within the sequence between the putative “−10” and “−35” promoter elements predicted by Katzen *et al.*^11^.

### Identification of the transcription initiation site and the RNA polymerase binding region in *gumB* promoter

As shown in Fig. 5C, the *gumB* promoter elements predicted in strain B-1459 are unusual as the spacer between the predicted “−35” and “−10” elements is 43 bp, which is much larger than the optimum 17 ± 1 bp^20^. An alignment of the upstream sequences of *gumB* genes in the strains ATCC33913, 8004, B100 and B-1459^6^,^7^,^8^,^11^ revealed that the “−31” nucleotide G from the predicted translation start codon ATG in strain B-1459 is absent in the other three strains. In addition, three nucleotides in strain 8004 differ from those in strain B-1459 (Supplementary Fig. 1). This prompted us to characterize the *gumB* promoter of strain 8004. First, a standard 5′-RACE method was carried out to determine its TIS. After mRNA was reverse transcribed into cDNA with *gumB* sequence-specific primer, an anchor sequence was added. The obtained tailored cDNA was then amplified by PCR with the nested gene-specific primers and the anchor-specific primer and cloned into the vector pMD19-T, resulting in recombinant plasmids for sequencing analysis. A high-quality sequence was obtained from 70 recombinant clones, 64 of which showed the same transcriptional start nucleotide at the position of the “−190” nucleotide C upstream of the translation start codon ATG predicted in strain B-1459, indicating that this “−190” nucleotide C is the TIS of the *gumB* operon in strain 8004 (Fig. 5C). From this TIS, the “−10” (CATAGT) and “−35” (TTGCGA) elements in the *gumB* promoter region of strain 8004 were defined, and the spacer between the two elements was found to be 16 bp (Fig. 5C). Second, we took a close look at the sequence of strain 8004 and found that there was an ATG at a position of 133 bp downstream of the determined TIS (Fig. 5C), which is also an in-frame codon of *gumB* ORF. Therefore, we propose that this ATG may be the translation start codon of *gumB* in strain 8004.

We also determined the sequence of the RNA polymerase (RNAP) binding region in the *gumB* promoter of strain 8004 using a DNase I footprinting assay on the 330-bp FAM-labelled DNA fragment. As the *gumB* promoter-*gusA* transcriptional fusion reporter analysis showed that the *gumB* promoter could be expressed in *E. coli* and *Xcc* RNAP is not available, *E. coli* RNAP holoenzyme was used. The result revealed that the RNAP protected a 70-bp DNA sequence extending from −41 to +29, relative to the TIS determined in this study (Fig. 5C, Supplementary Fig. 2), indicating that the interaction between the RNAP holoenzyme and *gumB* promoter in the transcription initiation complex involves this 70-bp DNA sequence.

### HpaR1 enhances *gumB* transcription *in vitro*

**Figure 6:**
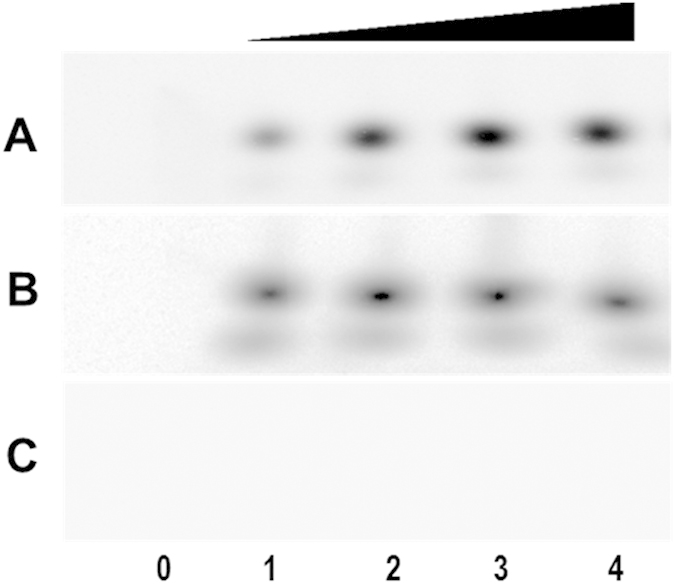
HpaR1 enhances the transcription of *gumB* promoter *in vitro*. (**A**) RNA was generated from a 687-bp template DNA fragment extending from −345 to +342 relative to the transcriptional initiation site (TIS) of the *gumB* promoter using *E. coli* RNA polymerase (RNAP) holoenzyme. (**B**) A template DNA fragment containing the *hrpG* promoter was used as a control. (**C**) A 329-bp template DNA fragment extending from +13 to +342 relative to the TIS of *gumB* promoter was also used as a control. 2 nM template DNA was incubated with a series of amounts of His_6_-HpaR1 protein before beginning transcription by the addition of 0.05 U of RNAP. Transcription products were then run on a 5% denatured polyacrylamide gel containing 7 M urea in 1× TBE electrophoresis buffer. Lane 0, template DNA alone; Lane 1, template DNA with RANP; Lanes 2–4, template DNA with RANP and 10, 15 and 20 nM His_6_-HpaR1.

The fact that HpaR1 binds to the promoter of *gumB* suggests that HpaR1 may directly regulate *gumB* transcription. To validate this, we performed an *in vitro* transcription assay. The assay was carried out using a 678 bp template DNA fragment extending from −345 to +342 relative to the TIS of the *gumB* promoter of strain 8004 and the RNAP holoenzyme from *E. coli*. The result showed that a certain amount of *gumB* transcripts could be generated without HpaR1 protein; however, the *gumB* transcription level was significantly increased when HpaR1 protein was added to the reaction (Fig. 6A), suggesting that HpaR1 could enhance *gumB* transcription *in vitro*. In this experiment, a template DNA fragment containing the *hrpG* promoter and a 329-bp DNA fragment extending from +13 to +342 relative to the TIS of *gumB* promoter of strain 8004 were used as controls. As shown in Fig. 6B,C, RNA could be generated from the *hrpG* promoter-containing template, but the addition of HpaR1 did not alter its transcriptional level. No transcription occurred from a 329-bp template comprising an internal fragment of the *gumB* gene lacking any promoter sequences. These data demonstrate that HpaR1 specifically enhances the transcription of *gumB* promoter.

### Mutation analysis verification that HpaR1 activates *gumB* transcription via binding to its promoter

**Figure 7:**
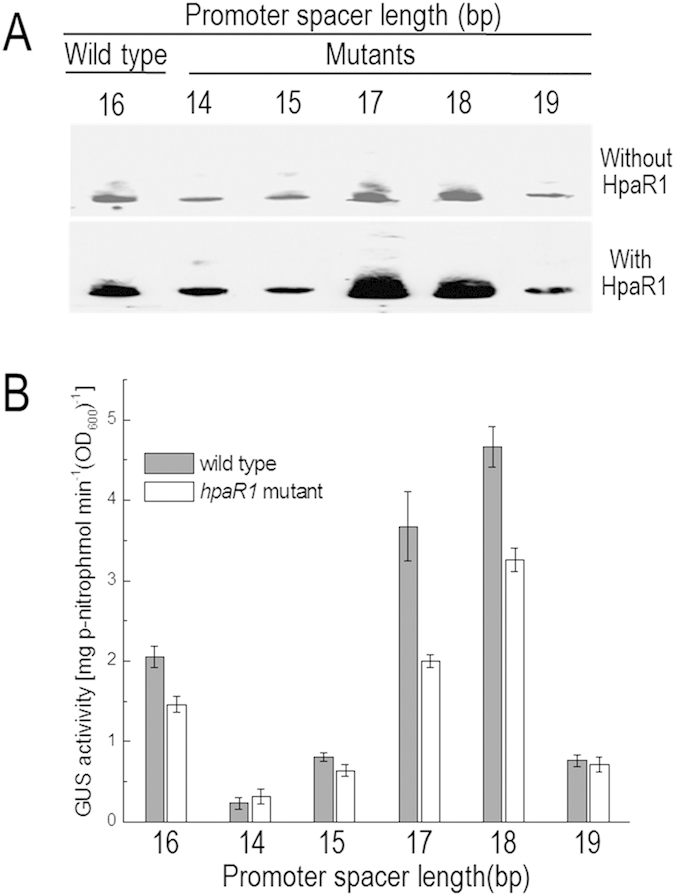
Shortening and lengthening the spacer between the −35 and −10 elements affect *gumB* promoter activity. (**A**) *In vitro* transcription assay. 1.15 nM DNA of 544–549 bp fragments of wild-type *gumB* promoter with a 16-bp-spacer or mutant *gumB* promoters with a 14-, 15-, 17-, 18- or 19-bp-spacer was incubated with 0.05 U of RNAP and 15 nM His_6_-HpaR1 protein if necessary. (**B**) GUS activity of *gumB* promoter-*gusA* reporters in the wild-type strain 8004 and the *hpaR1* mutant strain 2736nk. GUS activity was measured after the bacterial cells were cultured in NY medium containing 2% glucose for 24 h. Values given are the means ± standard deviations of triplicate measurements from a representative experiment; similar results were obtained in two other independent experiments.

The above results suggest that the spacer between the −10 and −35 elements of *gumB* promoter is 16 bp. As mentioned above, for transcriptional activation, the optimum length of the spacer is 17 ± 1 bp^20^. To gain some insight into *gumB* promoter and its activation by HpaR1, five *gumB* mutant promoters were constructed by deleting the −22 nucleotide “C” or the −23 and −22 nucleotides “TC” to shorten the spacer to 15 or 14 bp, or inserting 1, 2 and 3 nucleotide(s) at the position between the −22 and −21 to lengthen the spacer to 17, 18 and 19 bp, respectively (details below). *In vitro* transcription assay showed that the mutant promoters with a 14-, 15- or 19-bp-spacer generated a very small amount of transcripts, while the wild type (with a 16-bp-spacer) and the mutant promoters with a 17-or 18-bp-spacer produced a large amount of transcripts (Fig. 7A). These mutant promoters were also fused to promoterless *gusA* gene to construct transcriptional reporter plasmids (details below). GUS activity was measured after the reporter plasmids were introduced into the wild-type and *hpaR1* mutant strains. The result displayed that the promoters with a 16- to 18-bp-spacer produced significantly stronger GUS activities than those with a 14-, 15- or 19-bp-spacer (Fig. 7B). These data indicate that the optimum spacer length for *gumB* transcription is 16 to 18 bp. A comparison of the data presented in Fig. 7 revealed that the promoters with a 17- and 18-bp-spacer possess much stronger activity, compared to the wild-type promoter. Furthermore, both the *in vitro* transcription assay and the transcriptional reporter plasmid analysis revealed that HpaR1 enhanced significantly the transcription of these promoters (Fig. 7), further indicating that HpaR1 activates the transcription of *gumB*.

**Figure 8:**
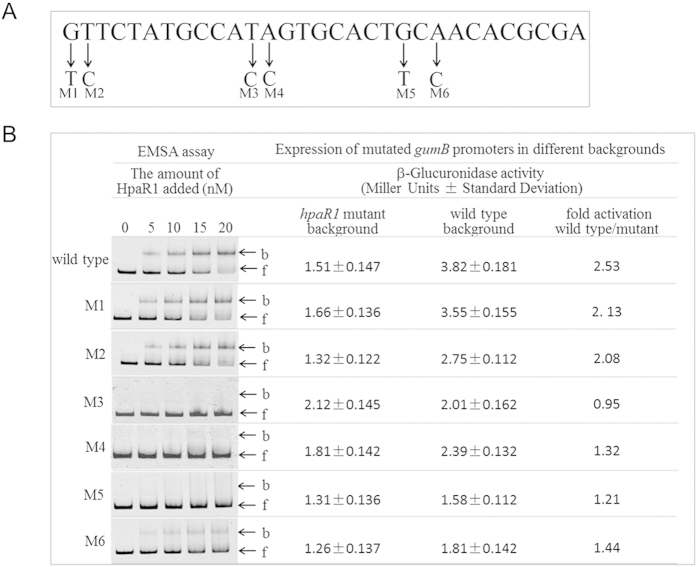
Point mutation analysis of the HpaR1 binding sequence in *gumB* promoter. (**A**) Six nucleotides were randomly chosen to be mutated and the resulting mutant fragments were designated as M1-M6. (**B**) FAM-labelled 287-bp *gumB* promoter DNA fragments (1.0 nM) with variant point mutations (M1-M6) or without a mutation (wild type) were incubated with increasing amounts of His_6_-HpaR1 protein (0, 5, 10, 15 and 20 nM) for 20 min at 30 °C before the electrophoretic mobility shift assay. The activity of these mutant promoters was detected by constructing promoter-*gusA* transcriptional fusion reporter plasmids and comparing their GUS activities in wild-type and *hpaR1* mutant backgrounds. b, bound DNA; f, free DNA.

To further verify the binding of HpaR1 to *gumB* promoter, six nucleotides within the HpaR1-binding sequence determined by footprint assay were selected arbitrarily to create point mutations by site-directed mutagenesis (details below) (Fig. 8A). The obtained labelled 287-bp (from −94 to +193, relative to the TIS) *gumB* promoter fragments carrying a single point mutation at the selected nucleotides were incubated with His_6_-HpaR1 and then analyzed by EMSA. The results showed that four of the point mutations (M3-M6) clearly diminished the binding of HpaR1, although two of them (M1 and M2) did not (Fig. 8B). To determine the activity of these *gumB* mutant promoters, promoter-*gusA* transcriptional fusion reporter plasmids were constructed and introduced by triparental conjugation into the *hpaR1* mutant strain and wild-type strain, respectively (details below). As shown in Fig. 8B, the four mutant promoters (M3-M6) that declined the binding of HpaR1 produced similar GUS activities in the wild-type background and *hpaR1* mutant background. The two mutant promoters, M1 and M2, that were still associated with HpaR1 produced about twofold higher GUS activities in the wild-type background compared to that produced in the *hpaR1* mutant background. These results further demonstrate that HpaR1 binds to the promoter of *gumB* and that a substitution of some nucleotides in the binding sequence can impede HpaR1 binding and the activation of *gumB* transcription.

### HpaR1 enhances RNA polymerase binding *in vitro*

**Figure 9:**
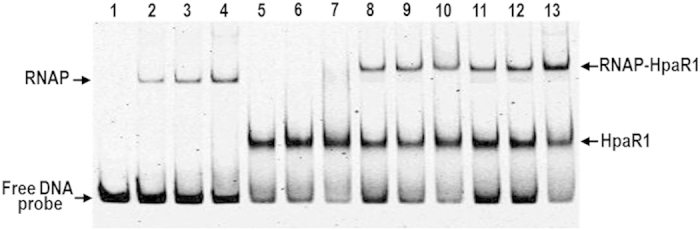
HpaR1 enhances RNA polymerase (RNAP) binding to the *gumB* promoter, as revealed by an electrophoretic mobility shift assay. The gel shift assay was carried out using 1.0 nM of 529-bp FAM-labelled DNA probe of the *gumB* promoter. RNAP or/and HpaR1was/were added as follows: lanes 1, 2, 3 and 4 with 0, 0.05, 0.1 and 0.2 U of RNAP without HpaR1; lanes 5, 6 and 7 with 12.5, 15 and 17.5 nM of HpaR1 without RNAP; lanes 8, 9 and 10 with 12.5, 15 and 17.5 nM of HpaR1 and 0.05 U of RNAP; lanes 11, 12 and 13 with 0.05, 0.1 and 0.2 U of RNAP and 12.5 nM of HpaR1.

The above data demonstrate that HpaR1 binds to a 31 nucleotide-sequence encompassing the “−10” element and the TIS of *gumB* promoter, and positively regulates *gumB* transcription. To gain an insight into the mechanism by which HpaR1 regulates the expression of *gumB*, we further employed the EMSA to test whether HpaR1 has an impact on the binding of RNAP to the *gumB* promoter. As shown in Fig. 9, a single shifted complex was formed and its intensity was enhanced along with the increase of either RNAP (lanes 2, 3 and 4: 0.05, 0.1 and 0.2 U) or HpaR1 (lanes 5, 6 and 7:12.5, 15 and 17.5 nM); while, on the contrary, the free *gumB* promoter DNA signal was weakened, indicating that the binding of RNAP or HpaR1 to *gumB* promoter was enhanced when the amount of either RNAP or HpaR1 was increased. In the presence of both RNAP and HpaR1, a new complex was formed, which is suggestive of a complex of RNAP and HpaR1 protein molecules bound to the *gumB* promoter DNA (Fig. 9, lanes 8–13). The intensity of these RNAP-HpaR1-*gumB* promoter complexes (Fig. 9, lanes 8–10) were significantly stronger than that of RNAP-*gumB* promoter complex (Fig. 9, lane 2), although they contained the same amount of RNAP (0.05 U). Moreover, the intensity of the RNAP-HpaR1-*gumB* promoter complexes was enhanced along with the increase in RNAP (Fig. 9, lanes 11–13), suggesting that more *gumB* promoter DNA molecules were recruited to the RNAP when HpaR1 was present. Taken together, these results demonstrate that HpaR1 can enhance *gumB* transcription *in vitro* by facilitating RNA polymerase binding.

## Discussion

Bacteria have evolved various mechanisms to accurately modify their gene expression to complete their life cycle and meet the challenge of micro-environmental changes. One of the main mechanisms is the use of helix-turn-helix transcriptional regulator proteins such as the GntR family. GntR family members are widely distributed in bacteria and their number in different bacteria varies from several to dozens^17^. *Xcc* contains only six predicted GntR members. Our previous work showed that five of them could be mutated to give little growth effect and one, HpaR1, is a *hrp*-associated regulator^14^. Herein, we have demonstrated that HpaR1 is a regulator of diverse cellular processes. In addition to controlling the expression of the *hrp* genes that are responsible for the hypersensitive reaction and pathogenicity, HpaR1 also regulates the production of extracellular enzymes and xanthan gum, and cell motility, as well as tolerance to various stresses. The GntR family of transcriptional regulators is named after the gluconate operon transcriptional repressor GntR of *Bacillus subtilis*, which was previously described by Haydon and Guest^21^. As reviewed by Hoskisson and Rigali^17^, GntR members have been found to play important roles in regulating many diverse biological processes, including primary metabolism, motility, development, antibiotic production and resistance, and plasmid transfer, as well as virulence. More recently, GntR members have also been demonstrated to be involved in the biofilm formation of *E. coli, Enterococcus faecalis* and *Listeria monocytogenes*^22^,^23^,^24^ and the EPS production of *Streptomyces* sp.^25^, although how they regulate their target genes remains unclear. Most of these diverse biological processes are regulated by different GntR members. To the best of our knowledge, HpaR1 is the first reported GntR member with a global regulatory function in plant bacteria. It was previously reported that the GntR-like protein NorG is a global regulator of the human pathogen *Staphylococcus aureus*, which directly or indirectly controls the expression of a wide range of genes, as revealed by transcriptional profiling microarray analysis^26^. The GntR member GfcR was demonstrated to regulate negatively the expression of diverse membrane-associated transporters and bacterial drug resistance in *Mycobacterium smegmatis*, a fast-growing nonpathogenic mycobacterium^27^.

To investigate the regulatory mechanisms of HpaR1, we began with the regulation of xanthan gum production. We showed that HpaR1 positively regulates the expression of the *gumB* operon that directs the *gum* cluster of genes, which is responsible for xanthan production. Although the *gumB* promoter could express at a certain level without HpaR1 protein *in vitro*, the addition of HpaR1 enhances significantly its transcription. This is consistent with the observations that the *hpaR1*-deficient mutant could still produce a certain amount of xanthan, but significantly less compared to that produced by the wild-type strain (Fig. 1A), and that the *gumB* in the *hpaR1*-deficient background could still express at a certain level, but its transcription was significantly weaker compared to that in the wild-type background (Fig. 1B,C). These results reveal that HpaR1 enhances *gumB* expression and xanthan gum production, although it is not absolutely essential for *gumB* expression and xanthan production.

DNase I footprint analysis revealed that HpaR1 and RNAP could specifically bind to a 31-bp and a 70-bp sequence, respectively, which extend from −21 to +10 and −41 to +29 relative to the TIS of *gumB* promoter. As HpaR1 acts as a positive regulator of *gumB* transcription, it is atypical that it binds to such a position. Generally, bacterial transcription activators bind the positions that are upstream of or overlapping the −35 promoter element, while repressors bind the spacer between the −35 and −10 elements^28^,^29^. There is, however, an exception to these general mechanisms, as it has been uncovered that a number of MerR family transcriptional activators bind to a region between or overlapping the −35 and −10 elements of their target promoters. For instance, MerR activates the transcription of *merT* by binding to a DNA sequence extending from −40 to −9 relative to the TIS of *merT*^30^. There are two common features in the target promoters of such MerR activators. One is that the length of the spacer between the −10 and −35 elements is 19 bp, which is greater than the optimum 17 ± 1 bp^31^, and the other is that the bound DNA region contains a dyad symmetrical sequence^30^. It has been proposed that the MerR members bind to the spacer sequence and twist or bend the DNA to reorientate the −10 and −35 elements so that they can be bound by RNAP^28^,^30^. A recent report suggested that GrlA in *E. coli* may use a similar mechanism to activate *LEE1* P1 promoter, but the region between the −35 and −10 elements in *LEE1* P1 promoter is 18 bp and does not contain a dyad symmetrical sequence^32^. A dyad symmetrical sequence also exists within the HpaR1-binding region, but the spacer between the −35 and −10 elements in *gumB* promoter is 16 bp (Fig. 5C). Shortening the spacer to 15 and 14 bp or lengthening it to 19 bp could almost inactivate the transcription of the promoter, while lengthening the spacer to 17 and 18 bp led to a significant increase of the promoter activity (Fig. 7), indicating that the optimum length of *gumB* promoter is 17 and 18 bp. In addition, HpaR1 could enhance the transcription of not only the wild-type promoter but also the mutant promoters with a 17- and 18-bp-spacer (Fig. 7). We speculate that without HpaR1, RNAP can still recognize and bind to the *gumB* promoter to initiate *gumB* transcription, but the recognition is weak, resulting in a low transcription level; however, HpaR1-binding can facilitate the RNAP-binding to the promoter, leading to a higher level of transcription. This speculation is supported by the results from the *in vitro* transcription assays.

As mentioned above, the GntR family consists of a large number of transcriptional regulators, which are widely distributed in bacteria and involved in various biological processes. However, up to now, only two binding sequences of YtrA subfamily members have been identified, i.e., the sequences recognized by YtrA in *ytrA* and *ywoB* promoter regions of *Bacillus subtilis*^33^,^34^. The sequences are 44 and 38 bp in length, respectively, and both of which contain a dyad symmetrical sequence^34^. Interestingly, the inverted repeat elements in both dyad symmetrical sequences are separated by 13 bp^34^, while the inverted repeat elements are linked by 1 bp in the HpaR1-binding sequence (Fig. 5C), suggesting that the HpaR1-binding sequence in *gumB* promoter may represent a novel type of YtrA subfamily targets. We believe that the findings in this work contribute to the understanding of the mechanisms by which the GntR family regulates the expression of their target genes. The interaction between HpaR1, RNAP and the *gumB* promoter represents an intriguing case study in the complexity of bacterial gene regulation. In addition to xanthan production, HpaR1 also positively regulates the expression of the *hrp* genes and genes involved in extracellular enzyme production and cell motility, as well as tolerance to various stresses. Further studies on the interactions between HpaR1 and these target genes will greatly enrich our knowledge, not only on the action of HpaR1, but also on the regulatory mechanisms of gene transcription.

## Methods

### Bacterial strains, plasmids and growth conditions

The bacterial strains and plasmids used in this study are listed in Supplementary Table 2. The *E. coli* strains were grown in Luria-Bertani medium^35^ at 37 °C, while the *Xcc* strains were grown at 28 °C in NYG medium (5 g of peptone, 3 g of yeast extract and 20 g of glycerol per litre)^36^ and in NY medium (NYG medium but without glycerol). Antibiotics were added at the following concentrations as required: kanamycin (Kan) at 25 μg ml^–1^; rifampicin (Rif) at 50 μg ml^–1^; ampicillin (Amp) at 100 μg ml^–1^; spectinomycin (Spc) at 50 μg ml^–1^ and tetracycline (Tet) at 5 μg ml^–1^ for *Xcc* and 15 μg ml^–1^ for *E. coli*.

### Motility assay

To test swarming motility, an overnight culture (OD_600_ of 1.0) of each *Xcc* strain was inoculated onto NY plates containing 2% glucose and 0.6% agar using a toothpick, and then incubated at 28 °C for 3 days. To detect swimming motility, the bacterial cells were stabbed into 0.28% agar plates composed of 0.03% Bacto peptone and 0.03% yeast extract followed by incubation at 28 °C for 4 days. The diameters of the area occupied by the bacterial cells were measured and these values were used to indicate the motility of the *Xcc* strains. The experiment was repeated at least three times.

### Extracellular enzyme activity and the xanthan gum assay

To estimate quantitatively the activity of the extracellular enzymes endoglucanase (cellulase), amylase, pectate lyase and protease, *Xcc* strains were cultured in NYG medium for 12 h. For endoglucanase, 10 μl of enzyme-containing extracts was added to 200 μl of indicator buffer containing 1% (wt/vol) carboxymethylcellulose (CMC, Sangon, Shangshai, China) as the substrate. The reactions were carried out for 30 min at 28 °C. The released reducing sugars were measured as D-glucose equivalents, as described by Miller^37^. One unit (U) of the endoglucanase activity was defined as the amount of enzyme releasing 1 μmol of reducing sugar per minute. Amylase activity quantification was conducted in the same way as for the endoglucanase measurement, except that the substrate 1% (wt/vol) CMC was replaced by 1% (wt/vol) starch solution. For pectate lyase, the activity was determined by measuring the increase in the absorbance at 235 nm of polygalacturonic acid (PGA) using a modification of the method described by Collmer and associates^38^, whereby 100 μl of enzyme-containing extracts in 100 mM Tris-HCl (pH 9.0) containing 500 μM CaCl_2_ and 0.2% (w/v) PGA were incubated at 30 °C for 30 min. The reaction was stopped by the addition of 20 μl of 0.35 M HCl. One unit of pectate lyase activity was defined as the amount of enzyme that produced 1 μmol of unsaturated galacturonide per minute. For extracellular protease activity, the method described by Swift and associates^39^ was used. To estimate xanthan gum production, the strains were cultured in 100 ml NY liquid medium containing 2% (w/v) glucose at 28 °C with shaking at 200 rpm for 3 d. Xanthan gum was precipitated from the culture supernatant with ethanol, then dried and weighed, as described by Tang and associates^40^.

### Stress tolerance assay

The well-established and widely used minimal inhibitory concentration (MIC) method^41^ was employed to test the resistance of the *Xcc* strains to several environmental stresses, including osmotic challenge (D-sorbitol), sodium dodecyl sulphate (SDS), the organic solvent phenol and heavy metal salts (CoCl_2_, CdCl_2_ and ZnSO_4_) stress. Briefly, *Xcc* strains were cultured to an OD_600_ of 0.6 and diluted; then 100 μl of the diluted culture was plated on NYG plates supplemented with different concentrations of each reagent, respectively. The surviving colonies on the plates were counted after three days of incubation at 28 °C.

### DNA and RNA manipulations

The DNA manipulations followed the procedures described by Sambrook *et al.*^42^. Conjugation between the *Xcc* and *E. coli* strains was performed as described by Turner *et al.*^43^. The restriction endonucleases, T4 DNA ligase and *pfu* polymerase were provided by Promega (Shanghai, China). The total RNAs were extracted from the cultures of the *Xcc* strains using a total-RNA extraction kit (Promega) according to the manufacturer’s instructions.

To assay the transcription level of *gumB*, real-time quantitative PCR (qRT-PCR) was carried out as previously described^44^. qRT-PCR was conducted with the total RNA extracted from the *Xcc* strains grown in NY medium containing 2% glucose for 12 and 48 h. The synergy brand (SYBR) green-labelled PCR fragments were amplified using the primer set gumNF/R (Supplementary Table 1), which was designed from the transcribed region of *gumB*. The relative mRNA level was calculated with respect to the level of the corresponding transcript in the wild-type strain 8004 (equalling 1). The expression level of the 16S rRNA gene was used as an internal standard. The qRT-PCR tests were performed in triplicate.

### Determination of transcription initiation site

To determine the transcription initiation site (TIS) of the *gumB* gene, the 5′-RACE method was carried out with the *gumB* sequence-specific primer gumRTP1-3 (Supplementary Table 1). The assay was performed as previously described^15^. Briefly, total cellular RNA was extracted from the *Xcc* strains grown in NY medium to an OD_600_ of 1.0. cDNA fragments were obtained using the 5′-RACE kit (Invitrogen Life Technologies, San Diego, CA, USA), and the PCR products were cloned into the pMD19-T vector and sequenced.

### Electrophoretic mobility shift assay (EMSA)

To obtain the HpaR1 protein, *E. coli* strain JM109/pQE-30-2736 expressing HpaR1 with a 6 × His tag on its N-terminus was grown to an optical density at OD_600_ of 0.6, and then induced by the addition of 1.0 mM IPTG. After the culture was grown for a further 4 h, the fused protein was purified using Ni-NTA resin^14^. The purified protein His_6_-HpaR1 was mixed with a 529-bp DNA fragment containing the putative *gumB* promoter region, which was amplified by PCR using the FAM-labelled primer set Pgum-2F/G (Supplementary Table 1). A control DNA fragment was amplified from the promoter sequence of *XC_1045* using the primers 1045F and 1045R (Supplementary Table 1).The labelled DNA fragment (1.0 nM) was incubated with purified protein for 20 min at 30 °C in 20 μl (total volume) of binding buffer (20 mM Tris-HCl, 10 mM NaCl, 1 mM EDTA and 1 mM dithiothreitol, pH 8.0) containing 1 μg of sonicated salmon sperm DNA and 3 μg of bovine serum albumin. The samples were loaded onto a 6% polyacrylamide-Tris-borate-EDTA (TBE) gel, and visualized after electrophoresis.

### Dye primer-based DNase I footprint assay

The HpaR1 binding sites on DNA were determined by the dye primer-based DNase I footprint method, as described by Zianni *et al.*^45^. A 330-bp fragment encompassing bases −164 to +166 from the TIS of *gumB* promoter in *Xcc* strain NRRL B-1459, determined by Katzen *et al.*^11^, was amplified by PCR from the total DNA of *Xcc* strain 8004 with the primer set Pgum-3F/R (Supplementary Table 1). About 0.5 μM of 6-carboxyfluorescein (FAM)-labelled *gumB* promoter was incubated with various amounts of HpaR1 protein ranging from 0 to 30 μM in a binding buffer (5% glycerol, 20 mM Tris-Cl, pH 8.0, 50 mM KCl, 1 mM dithiothreitol, 100 μg/ml bovine serum albumin and 5 μg of shared DNA). The HpaR1-bound promoter region was then digested with DNase I to determine the region that it binds to. After several optimization experiments, the DNase I digestion was found to work best with 0.05 Kunitz units of DNaseI (New England BioLabs) per 20μl reaction mixture for 5 min at room temperature. The reaction was stopped with 0.25 M EDTA and extracted with phenol-chloroform-isoamyl alcohol (25:24:1). The DNA fragments were purified with a QIAquick PCR Purification kit (Qiagen) and eluted in 15 μl of distilled water. About 5 μl of digested DNA was added to 4.9 μl of HiDi formamide (Applied Biosystems) and 0.1 μl of GeneScan-500 LIZ size standards (Applied Biosystems). The samples were analyzed with a 3730 DNA Analyzer. The results were analyzed with GeneMarker software (Softgenetics). The assay was repeated at least three times and gave similar results. The RNAP (*E. coli* RNA polymerase holoenzyme) binding site was determined by a similar procedure.

### Site-directed mutagenesis

For nucleotide substitution in the HpaR1 binding sequence we first cloned a 546-bp region of the *gumB* promoter into the suicide plasmid pK18*mob*^46^ to make a recombinant plasmid pKgum. For this we used primer set Pgum-1F/R to amplify the region with *Eco*RI and *Bam*HI ends to facilitate the cloning. Site-directed mutagenesis was then carried out using a QuikChange^TM^ II Site-directed Mutagenesis Kit (Stratagene) with the recombinant plasmid pKgum as the template and the appropriate primer sets, which are listed in Supplementary Table 1. The plasmids with a point mutation obtained from the site-directed mutagenesis were verified by sequencing and used as templates for PCR amplification of the 287-bp (from −94 to +193, relative to the TIS) mutant *gumB* promoter fragments with the FAM-labelled primer pair Pgum-4F/R (Supplementary Table 1). The PCR products were purified and used for EMSA. To determine the activity of the obtained mutant *gumB* promoters, the plasmids with a point mutation were digested with *Eco*RI/*Bam*HI to release the 546-bp DNA fragments containing the mutant *gumB* promoters, which were then cloned into plasmid pL6*gus* (Supplementary Table 2).The resulting recombinant plasmids were introduced by triparental conjugation into *Xcc* strains for GUS activity assay, as described previously^40^. Similar site-directed mutagenesis was used for nucleotide insertion or deletion in the spacer between the “−10” and “−35” elements to lengthen or shorten the spacer of *gumB* promoter. The primer sets with corresponding insertions (1, 2 and 3 bp) or deletions (1 and 2 bp) were listed in Supplementary Table 1. The obtained plasmids carrying a *gumB* promoter with nucleotide insertion or deletion were digested with *Eco*RI/*Bam*HI to release the 544 to 549-bp DNA fragments containing the deletion or insertion mutant *gumB* promoters, and the fragments were used for *in vitro* transcription assays and construction of promoter reporter plasmids (Supplementary Table 2).

### *In vitro* transcription assay

An *in vitro* transcription assay of *gumB* promoter was performed using an experimental procedure modified from Friedman and O’Brian^47^. The promoter DNA fragment of *gumB* was generated by PCR amplification using the total DNA of the wild-type strain 8004 as the template and the primers gumivtF and gumivtR (Supplementary Table 1). The fragment includes the promoter region as well as the 209-bp coding region downstream of the annotated translation start site of the *gumB* gene in strain 8004. To remove imidazole, His_6_-HpaR1 was dialyzed against 200-time-volumes of Tris-HCl buffer (10 mM Tris-HC [pH 8.0] and 1 mM dithiothreitol [DTT]) at 4 °C. For the *in vitro* transcription, His_6_-HpaR1 was incubated for 30 min at room temperature in transcription buffer (40 mM Tris-HCl [pH 7.9], 6 mM MgCl_2_, 2 mM spermidine, 10 mM NaCl, 5 mM DTT, 5% glycerol, 50 mM KCl and 1 U of RNase inhibitor) containing 2 nM or 1.15 nM template DNA. Then, an NTP mixture (250 μM each of ATP, CTP and GTP; 20 μM UTP and 8 μM [α-^32^P]UTP [3,000 Ci/mmol, 10 mCi/ml] or 250 μM Biotin-16-UTP) and 0.05 U of *E. coli* RNA polymerase holoenzyme (sigma-saturated) was added to start the transcription. After incubation at 28 °C for 30 min, the reactions were terminated by the addition of one volume of 2× Loading Dye Solution (Fermentas Co.) and chilled on ice. After incubation at 70 °C for 10 min, the transcription products were run on a 5% polyacrylamide gel containing 7 M urea in 1× TBE electrophoresis buffer. The transcripts obtained were analyzed by a phosphorimager screen (Typhoon 9410; Amersham Biosciences, Piscataway, NJ, USA).

### GUS activity assay

GUS (*β-*Glucuronidase) activity was determined by measurement of the A_415_, using *ρ*-nitrophenyl-*β*-D-glucuronide as the substrate, as described by Henderson *et al.*^48^ after growth of the *Xcc* strains in medium.

## Additional Information

**How to cite this article**: Su, H.-Z. *et al.* Characterization of the GntR family regulator HpaR1 of the crucifer black rot pathogen *Xanthomonas campestris* pathovar *campestris*. *Sci. Rep.* **6**, 19862; doi: 10.1038/srep19862 (2016).

## Supplementary Material

Supplementary Data

## Figures and Tables

**Table 1:** Quantification of extracellular enzymes produced by *Xcc* strains*.

Strains	Protease (A_366_)	Endoglucanase (U)	Amylase (U)	Pectate lyase (U)
8004 (wild type)	0.22 ± 0.02a	0.62 ± 0.07a	0.42 ± 0.05a	0.68 ± 0.04a
2736nk (*hpaR1* mutant)	0.12 ± 0.02b	0.45 ± 0.03b	0.32 ± 0.02b	0.32 ± 0.03b
C2736 (complemented strain)	0.24 ± 0.03a	0.68 ± 0.05a	0.45 ± 0.04a	0.65 ± 0.06a

*Data are the mean ± standard deviation of triplicate measurements; the different letters in each data column indicate significant differences at *P* = 0.01. The experiment was repeated twice and similar results were obtained.
