# Supplementary material for: Characterization of the GntR family regulator HpaR1 of the crucifer black rot pathogen Xanthomonas campestris pathovar campestris
Source: Sci Rep. 2016 Jan 28;6:19862. doi: 10.1038/srep19862 (PMC4730234; doi:10.1038/srep19862)
Supplement: Supplementary Data [file srep19862-s1.pdf]

Supplementary information

Title: Characterization of the GntR family regulator HpaR1 of the crucifer black rot pathogen Xanthomonas campestris pathovar campestris.

Authors: Hui-Zhao Su, Liu Wu, Yan-Hua Qi, Guo-Fang Liu, Guang-Tao Lu & Ji-Liang Tang

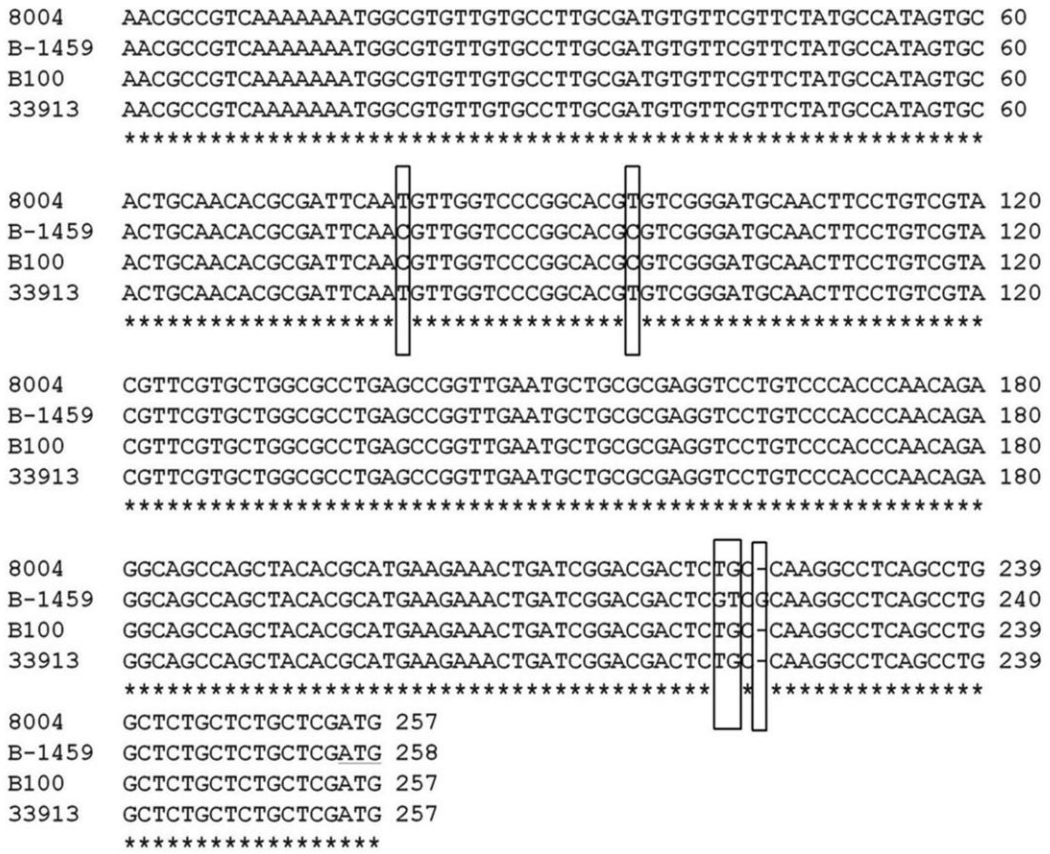

Supplementary Figure 1. Comparison of the 255-bp *gumB* promoter sequences of *Xcc* strains 8004, ATCC33913, B100 and B-1459. Rectangular squares indicate the bases in the strains differ from one another. The predicted translation start site ATG in strain B-1459 is underlined.

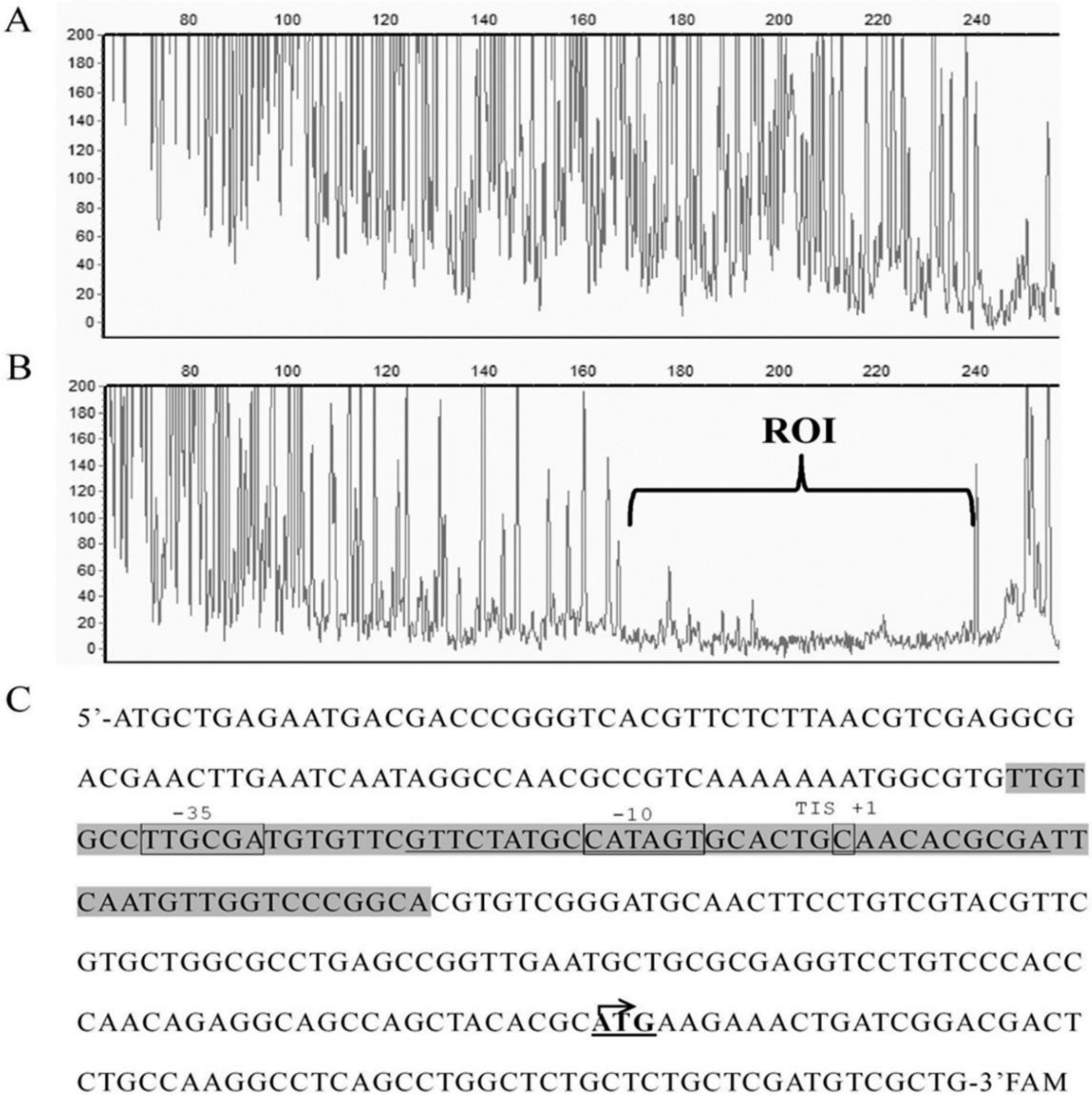

Supplementary Figure 2. Determination of the RNA polymerase binding region in the *gumB* promoter of *Xcc* strain 8004 by dye primer-based DNase I footprint assay. Electropherograms show the protection pattern of the *gumB* promoter after digestion with DNase I following incubation in the absence (**A**) or presence (**B**) of 1 U *E. coli* RNA polymerase (RNAP). ROI, region of interest. (**C**) DNA sequence of 330-bp fragment (labelled with FAM at the 3' end) used in this assay. The protected region by RNAP, i.e. the RNAP-binding region, is shown in the grey background (-41 to +29 relative to the transcription initiation site). The HpaR1 binding site is underlined. The transcription initiation site is indicated by a square, and the putative translation start codon ATG is underlined. The -35 and -10 elements are boxed.

Supplementary Table 1. Primers used in this study

Primer	Nucleotide sequence (5′→3′)	The amplified segment or the primer location
Pgum-1F<br>Pgum-1R	ACAGTTGAATTCGGCCCATGTCGCAAGACGTG<br>ACAGTTGGATCCCATCGAGCAGAGCAGAGCCAG	546-bp DNA sequence upstream of the start codon of *gumB* of *Xcc* strain B-1459 (include ATG), used for construction of reporter plasmids and site-directed mutagenesis.
Pgum-2F<br>Pgum-2R	AACAACAGGCCCATGTCGCA<br>CATCGAGCAGAGCAGAGCCAG	529-bp DNA sequence upstream of the start codon of *gumB* of *Xcc* strain B-1459 (include ATG), used for EMSA, FAM-labeled.
Pgum-3F<br>Pgum-3R	ATGCTGAGAATGACGACCCGGG<br>CAGCGACATCGAGCAGAGCAGA	330-bp DNA fragments spanning nucleotides -164 to +166 from the transcription initiation site of the *gumB* promoter region of *Xcc* strain B-1459, used for Dye primer-based DNase I footprint assay, FAM-labelled.
Pgum-4F<br>Pgum-4R	GTCGAGGCGACGAACTTGAA<br>CATCGAGCAGAGCAGAGCCAG	287-bp DNA sequence upstream of the start codon of *gumB* of *Xcc* strain B-1459 (include ATG), used for EMSA, FAM-labelled.
gumivtF<br>gumivtR	AACAACAGGCCCATGTCGCAAG<br>CGCTCCAGATCGTCGATCTGAAAC	678-bp DNA fragments including the promoter region and 209-bp coding region downstream of the start codon of *gumB* of *Xcc* strain 8004, used for *in vitro* transcription assay.
gumivt-1F<br>gumivt-1R	AATGTTGGTCCCGGCACGTG<br>CGCTCCAGATCGTCGATCTGAAAC	329-bp DNA fragments including 209-bp coding region downstream of the start codon of *gumB* of *Xcc* strain 8004, without the -35 and -10 promoter elements, used as a control in *in vitro* transcription assay.
gumNF<br>gumNR	GATCTGTTGCTGGTGAAGGTGTTTC<br>CTGAAGGTAGCCAGCGCGATA	171-bp internal fragment of *gumB*, used for qRT-PCR.
RTPgum1<br>RTPgum2<br>RTPgum3	TCGTCCACTGCACCAGTGACCGTGA<br>TGACCGTTCTGGTCGATGCGGACCT<br>CATCGAGCAGAGCAGAGCCAG	located in *gumB* ORF, used for 5′-RACE
PM1F<br>PM1R	CCTTGCGATGTGTTCTTTCTATGCCATAGTGC<br>GCACTATGGCATAGAAAGAACACATCGCAAGG	Used for creating M1 DNA fragment with point mutation.
PM2F<br>PM2R	CCTTGCGATGTGTTCGCTCTATGCCATAGTGCAC<br>GTGCACTATGGCATAGAGCGAACACATCGCAAGG	Used for creating M2 DNA fragment with point mutation.
PM3F<br>PM3R	GCGATGTGTTCGTTCTATGCCACAGTGCACTGCAACACGCG<br>CGCGTGTTGCAGTGCACTGTGGCATAGAACGAACACATCGC	Used for creating M3 DNA fragment with point mutation.
PM4F<br>PM4R	GATGTGTTCGTTCTATGCCATCGTGCACTGCAACACGCG<br>CGCGTGTTGCAGTGCACGATGGCATAGAACGAACACATC	Used for creating M4 DNA fragment with point mutation.
PM5F<br>PM5R	CTATGCCATAGTGCACTTCAACACGCGATTCAATG<br>CATTGAATCGCGTGTTGAAGTGCACTATGGCATAG	Used for creating M5 DNA fragment with point mutation.
PM6F<br>PM6R	CTATGCCATAGTGCACTGCCACACGCGATTCAATGTTGG<br>CCAACATTGAATCGCGTGTGGCAGTGCACTATGGCATAG	Used for creating M6 DNA fragment with point mutation.

1045F<br>1045R	CGACGACGTTTGAGCACGCCGTCCTAT<br>AAGCCGCTGTTCATCTGGTCCGCCA	311-bp DNA sequence including the promoter region and 122-bp coding region of the gene *XC_1045*, used for EMSA, FAM-labelled.
3077F<br>3077R	TCTCACTCTGTCTTGCAAACTGCGA<br>AATGGGCATCGAAAACCAGAAGC	890-bp DNA fragment including the promoter region and coding region of *hrpG* of *Xcc* strain 8004, used as a control in *in vitro* transcription assay.
16SF<br>16SR	GCCTAACACATGCAAGTCGAACGGC<br>AATATTCCCCACTGCTGCCTCCCG	325-bp DNA fragment of the 16S rDNA sequence, used for qRT-PCR.
PD1F<br>PD1R	TGTGCCTTGCGATGTGTTGTTCTATGCCATAGTGCACT<br>AGTGCACTATGGCATAGAACAACACATCGCAAGGCACA	Used for creating *gumB* mutant promoter with one nucleotide deletion.
PD2F<br>PD2R	TGTGCCTTGCGATGTGTGTTCTATGCCATAGTGCACT<br>AGTGCACTATGGCATAGAACACACATCGCAAGGCACA	Used for creating *gumB* mutant promoter with two nucleotides deletion.
PIN1F<br>PIN1R	TGTGCCTTGCGATGTGTTCCGTTCTATGCCATAGTGCACT<br>AGTGCACTATGGCATAGAACGGAACACATCGCAAGGCACA	Used for creating *gumB* mutant promoter with one nucleotide insertion.
PIN2F<br>PIN2R	TGTGCCTTGCGATGTGTTCCAGTTCTATGCCATAGTGCACT<br>AGTGCACTATGGCATAGAACTGGAACACATCGCAAGGCACA	Used for creating *gumB* mutant promoter with two nucleotides insertion.
PIN3F<br>PIN3R	TGTGCCTTGCGATGTGTTCTCAGTTCTATGCCATAGTGCACT<br>AGTGCACTATGGCATAGAACTGAGAACACATCGCAAGGCACA	Used for creating *gumB* mutant promoter with three nucleotides insertion.

Supplementary Table 2. Strains and plasmids used in this study

Strains or plasmids	Relevant characteristics	Reference or source
E. coli		
JM109	*RecA*1, *endA*1, *gyrA*96, *thi, supE*44, *relA*1△ (*lac-proAB*)/F' [*traD36, lacI*^{q}, *lacZ* △M15]	(1)
DH5α	Φ80△*lacZM*15 *recA1 endA1 deoR*	Gibco BRL, Life Technologies
JM109/pQE-30-2736	JM109 harbouring pQE-30-2736	(2)
X. campestris pv. *campestris*		
8004	Wild type, Rif^r	(3)
2736nk	As 8004, but *XC_2736*::pK18*mob*, non-polar effect. Rif^r, Kan^r	(2)
8004/pGUSgum	8004 harbouring pGUS*gum*, Rif^r Tet^r	This work
2736nk/pGUSgum	2736nk harbouring pGUS*gum*, Rif^r Kan^r Tet^r	This work
Plasmids		
pLAFR6	A promoterless derivative of pLAFR3, Tet^r	(4)
pRK2073	Helper plasmid, Tra+, Mob+, ColE1, Spc^r.	(5)
pQE-30	Expression vector, allowing the production of fusion proteins containing amino terminal 6xHis-tagged sequences. Amp^r	Qiagen, Germany
pQE-30-2736	pQE-30 containing a 360-bp fragment of *hpaR1* gene coding region.	(2)
pL6*gus*	pLAFR6 containing a 1,832-bp *gusA* ORF (excluding ATG), Tet^r	(6)
pGUSgum	pLAFR6 containing 546-bp promoter region of the gene *gumB* fused to the coding region of *gusA*, Tet^r	This work
pK18*mob*	pUC18 derivative, *lacZα* Kan^r, *mob* site. Suicide plasmid in *Xcc.*	(7)
pK*gum*	The suicide plasmid pK18mob containing 546-bp *gumB* promoter.	This work
pGUSgum$_{M1-6}$	pLAFR6 containing 546-bp *gumB* promoter with 6 different single mutated nucleotide, respectively, fused to the coding region of g*usA*, Tet^r	This work
pGUSgum$_{15}$	pLAFR6 containing the mutant *gumB* promoter with 1 nucleotide deletion in the spacer between -35 and -10 elements, fused to the coding region of *gusA*, Tet^r	This work
pGUSgum$_{14}$	pLAFR6 containing the mutant *gumB* promoter with 2 nucleotides deletion in the spacer between -35 and -10 elements, fused to the coding region of *gusA*, Tet^r	This work
pGUSgum$_{17}$	pLAFR6 containing the mutant *gumB* promoter with 1 nucleotide insertion in the spacer between -35 and -10 elements, fused to the coding region of *gusA*, Tet^r	This work
pGUSgum$_{18}$	pLAFR6 containing the mutant *gumB* promoter with 2 nucleotides insertion in the spacer between -35 and -10 elements, fused to the	This work

	coding region of *gusA*, Tet^r	
pGUSgum$_{19}$	pLAFR6 containing the mutant *gumB* promoter with 3 nucleotides insertion in the spacer between -35 and -10 elements, fused to the coding region of *gusA*, Tet^r	This work

References

1. Yanisch-Perron, C., Vieira, J. & Messing, J. Improved M13 phage cloning vectors and host strains: nucleotide sequences of the M13mp18 and pUC19 vectors. *Gene* **33**, 103–119 (1985).
2. An, S. Q. *et al.* Systematic mutagenesis of all predicted *gntR* genes in *Xanthomonas campestris* pv. *campestris* reveals a GntR family transcriptional regulator controlling hypersensitive response and virulence. *Mol. Plant-Microbe Interact.* **24**, 1027–1039 (2011).
3. Daniels, M. J. *et al.* Cloning of genes involved in pathogenicity of *Xanthomonas campestris* pv. *campestris* using the broad host range cosmid pLAFR1. *EMBO J.* **3**, 3323–3328 (1984).
4. Huynh, T. V., Dahlbeck, D. & Staskawicz, B. J. Bacterial blight of soybean: regulation of a pathogen gene determining host cultivar specificity. *Science* **245**, 1374–1377 (1989).
5. Leong, S. A., Ditta, G. S. & Helinski, D. R. Heme biosynthesis in *Rhizobium*. Identification of a cloned gene coding for delta-aminolevulinic acid synthetase from *Rhizobium meliloti*. *J. Biol. Chem.* **257**, 8724–8730 (1982).
6. Jiang, B. L. *et al.* The type III secretion effector XopXccN of *Xanthomonas campestris* pv. *campestris* is required for full virulence. *Res. Microbiol.* **159**, 216–220 (2008).
7. Schäfer, A. *et al.* Small mobilizable multi-purpose cloning vectors derived from the *Escherichia coli* plasmids pK18 and pK19: selection of defined deletions in the chromosome of *Corynebacterium glutamicum*. *Gene* **145**, 69–73 (1994).
